# Upper abdominal body shape is the risk factor for postoperative pancreatic fistula after splenectomy for advanced gastric cancer: A retrospective study

**DOI:** 10.1186/1477-7819-6-109

**Published:** 2008-10-10

**Authors:** Naoto Yamamoto, Takashi Oshima, Tsutomu Sato, Hirochika Makino, Yasuhiko Nagano, Shoichi Fujii, Yasushi Rino, Toshio Imada, Chikara Kunisaki

**Affiliations:** 1 Yokohama City University Medical Center, Gastroenterological Surgery, Yokohama, Japan; 2 Yokohama City University, Yokohama, Japan

## Abstract

**Background:**

Postoperative pancreas fistula (POPF) is a major complication after total gastrectomy with splenectomy. We retrospectively studied the effects of upper abdominal shape on the development of POPF after gastrectomy.

**Methods:**

Fifty patients who underwent total gastrectomy with splenectomy were studied. The maximum vertical distance measured by computed tomography (CT) between the anterior abdominal skin and the back skin (U-APD) and the maximum horizontal distance of a plane at a right angle to U-APD (U-TD) were measured at the umbilicus. The distance between the anterior abdominal skin and the root of the celiac artery (CAD) and the distance of a horizontal plane at a right angle to CAD (CATD) were measured at the root of the celiac artery. The CA depth ratio (CAD/CATD) was calculated.

**Results:**

POPF occurred in 7 patients (14.0%) and was associated with a higher BMI, longer CAD, and higher CA depth ratio. However, CATD, U-APD, and U-TD did not differ significantly between patients with and those without POPF. Logistic-regression analysis revealed that a high BMI (≥25) and a high CA depth ratio (≥0.370) independently predicted the occurrence of POPF (odds ratio = 19.007, p = 0.002; odds ratio = 13.656, p = 0.038, respectively).

**Conclusion:**

Surgical procedures such as total gastrectomy with splenectomy should be very carefully executed in obese patients or patients with a deep abdominal cavity to decrease the risk of postoperative pancreatic fistula. BMI and body shape can predict the risk of POPF simply by CT.

## Background

Gastrectomy with D2 lymph node dissection is an established procedure for the treatment of gastric cancer in Japan [1-3]. Japanese retrospective studies have shown that 20%–30% of patients with advanced cancer of the proximal stomach have nodal metastasis at the splenic hilum. Gastrectomy with dissection for these nodes can yield a 5-year survival of 20%–25%[4].

The most frequent major complication after total gastrectomy with extended dissection is pancreatic fistula [3,5-7]. European clinical trials have shown that pancreatic complications are a major cause of mortality after gastrectomy [8,9]. Moreover, postoperative pancreatic complications are difficult to treat and prolong hospitalization.

Total gastrectomy is a challenging procedure, even for experienced, skilled surgeons because deep sites around the esophageal hiatus or esophagojejunal anastomosis have to be dissected. The depth of the surgical sites is thought to correlate with the difficulty of total gastrectomy, but only a few studies have examined related factors [10-12].

This study was designed to evaluate the effects of abdominal shape at the umbilicus and the upper abdomen on short-term surgical outcomes, particularly the incidence of postoperative pancreas fistula (POPF) in patients undergoing total gastrectomy with splenectomy.

## Methods

### Patients

We retrospectively studied 50 consecutive patients with advanced cancer arising in the upper third of the stomach who underwent D2 or more extensive total gastrectomy with splenectomy between January 2004 and August 2006 at the Department of Surgery, Gastroenterological Center, Yokohama City University. All of the subjects were preoperatively confirmed to have gastric adenocarcinoma on histological examination of endoscopic biopsy specimens. The preoperative evaluation included a barium-swallow examination, an endoscopic examination with biopsy, and computed tomography (CT) in all patients. Abdominal and endoscopic ultrasonography were optional. Staging and lymph node dissection were performed as recommended by the Japanese Research Society for Gastric Cancer [13].

### Quantification of abdominal shape

**Figure 1:**
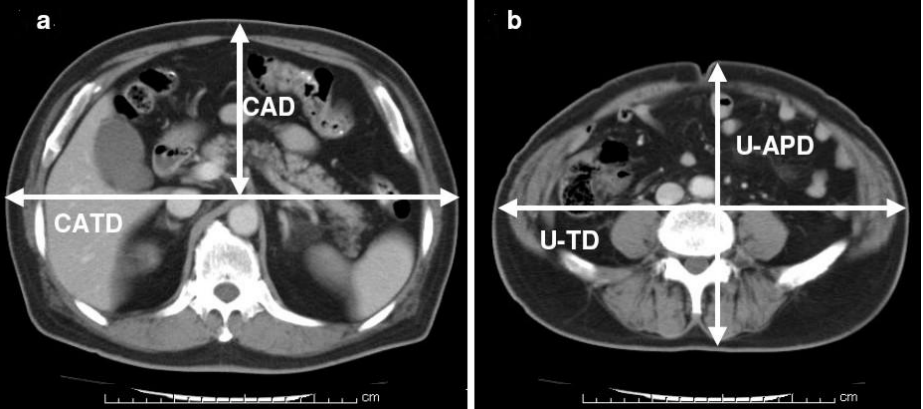
**Measurement of body shape**. Figures 1a and 1b represent the same patient's images who suffered POPF: a 73-year-old male (gastric cancer), 165 cm, 73 kg, BMI 26.8 kg/m2, CAD 13.1 cm, CATD 32.2 cm, CA-depth ratio 0.407, U-APD 20.0 cm, U-TD 29.0 cm.

All CT were obtained with patients in a supine position, using a helical CT scanner within 2 months before gastrectomy. The distance between the anterior abdominal skin and the root of celiac artery was defined as CAD. The distance of a horizontal plane at a right angle to CAD was defined as CATD. CAD and CATD were measured on CT at the level of the root of the celiac artery (Figure. 1a). We then calculated the CA depth ratio (CAD/CATD) to more morphologically describe body shape. The maximum vertical distance between the anterior abdominal skin and the back skin was defined as U-APD. The maximum horizontal distance of a plane at a right angle to U-APD was defined as U-TD. U-APD and U-TD were measured on CT scans at the level of the umbilicus (Figure. 1b).

Median U-APD, U-TD, CAD, CATD, and CA depth ratio were 19.0 cm (range 13.0–24.0), 29.0 cm (range 21.0–35.0), 10.1 cm (range 5.9–14.2), 29.5 cm (range 23.5–34.2), and 0.370 (range 0.218–0.473), respectively.

### Surgical Technique

After transection of the proximal side of the specimen (usually at the abdominal esophagus), the spleen and pancreatic tail is removed from the retroperitoneum. The lymph nodes along the splenic artery are removed, taking particular care to avoid injuring the pancreas. The splenic artery is divided at the end of the pancreatic parenchyma. The splenic vein is ligated and resected at the same level as the splenic artery. After removing the surgical specimen (including the stomach, greater and lesser omenta, and lymph nodes), the correct extent of the retroperitoneal dissection can be fully assessed. Reconstruction was routinely done using a Roux-en-Y technique with a stapler after total gastrectomy; a 25-mm circular stapler was usually used. All patients received antibiotic prophylaxis for the same period. Two or more closed-type drains are routinely applied in the left subphrenic space and around the stump of duodenum in all patients. Drains are removed after the 7^th ^post operative day if there isn't the incidence of intraabdominal complication such as anastomotic leakage or POPF. In case of having intraabdominal infectious complication, we changed drains under radiographic examination and lavaged the cavity through the drains once or twice a day.

### Definition of Postoperative Pancreatic Fistula (POPF)

A case of POPF had to satisfy the criteria for the postoperative pancreatic fistula after pancreaticoduodenectomy: Output via an operatively placed drain of any measurable volume of drain fluid on or after postoperative day 3, with amylase content greater than 3 times the upper normal serum level [14].

### Statistical Analysis

We reviewed the patients' medical charts and surgical records to obtain the following information: sex (female or male), body mass index (BMI), age (years, <60 or ≥60), operation time (minutes, <300 or ≥300), and volume of bleeding (ml, <500/≥500, <1000/≥1000). Variables of body shape were classified as follows: BMI (kg/m^2^, <25, or ≥25)[15], U-APD (cm, <19 or ≥19), U-TD (cm, <29 or ≥29), CAD (cm, <10 or ≥10), CATD (cm, <29 or ≥29), and CA depth ratio (<0.370 or ≥0.370). Variables of body shape except for BMI were divided into two groups by median because biologically meaningful cutoff points could not be defined. Preoperative hemoglobin and albumin levels are expressed as means ± SD and were analyzed with Student's t-test. Frequencies were analyzed using the χ^2 ^test or Fisher's exact test. Two-sided p values of less than 0.05 were considered to indicate statistical significance. All of the factors that were significant in the univariate analysis were included in the logistic regression analysis. All analyses were performed using the SPSS program version 11.0.1J for Windows (SPSS Inc., Chicago, IL). This study was approved by our institutional review board.

## Results

### Clinicopathological characteristics of patients

Pancreatic fistula was diagnosed in 7 of the 50 patients (14.0%). There was no postoperative death due to pancreatic fistula within 30 days or during the hospital stay. The median age of the patients was 66 years (range 39–82 years), and there were 42 (84%) men and 8 (16%) women (Table 1). All patients underwent total gastrectomy and pancreas-preserving splenectomy with D2 or more extended lymph node dissection. The mean operation time was 346 min (range 197–640). There was no difference between the patients with POPF and those without POPF with respect to gender, age, preoperative serum albumin level, hemoglobin level, operation time, or the volume of bleeding (Table 1).

**Table 1:** Comparison of clinicopathologic characteristics according to the presence or absence of postoperative pancreatic fistula

	POPF(-)	POPF(+)	P value
Gender, Female/male	8/35	0/7	0.579
Age (yr), <60/≥60	20/23	2/5	0.444
Albumin (g/dl)	3.9 ± 0.5	3.9 ± 0.5	0.794
Hemoglobin (g/dl)	12.7 ± 1.8	13.0 ± 2.7	0.369
Operation time (min), <300/≥300	16/27	3/4	>0.999
Volume of bleeding (ml), <500/≥500, <1000/≥1000	24/14/5	3/2/2	0.484

### Incidence of POPF according to the surgeons' experience

Three different surgeons operated on patients within the study group. According to the numbers of previous gastrectomy combined with splenectomy performed by each surgeon (<20 cases vs. ≥20 cases), the surgeons' experience was unrelated to the incidence of POPF; POPF occurred in 4 of 27 patients who underwent gastrectomy by one inexperienced surgeons (<20 cases) and in 3 of 23 patients who underwent gastrectomy by two experienced surgeons (≥20 cases) (P > 0.9999).

### Correlation of abdominal shape and body mass index with POPF

Body shape significantly differed between patients with POPF and those without POPF. POPF was significantly associated with a higher BMI, longer CAD, and higher CA depth ratio. However, the presence of POPF was unrelated to CATD, U-APD, and U-TD (Table 2).

**Table 2:** Comparison of BMI and body shape according to the presence or absence of postoperative pancreatic fistula

	POPF(-)	POPF(+)	P value
Body mass index (kg/m^2^), <25/≥25	39/4	3/4	0.009
CAD (cm), <10/≥10	25/18	1/6	0.045
CATD (cm), <29/≥29	23/20	2/5	0.417
U-APD (cm), <19/≥19	23/20	2/5	0.417
U-TD (cm), <29/≥29	29/14	3/4	0.234
CA depth ratio, <0.370/≥0.370	36/7	2/5	0.006

### Logistic-regression analysis for the prediction of POPF

The three factors (BMI, CAD, and CA depth ratio) that were significantly associated with POPF in the univariate analysis were entered into a logistic-regression analysis. BMI and CA depth ratio were found to independently predict the occurrence of POPF (Table 3).

**Table 3:** Predictive factors for POPF as assessed by logistic-regression analysis

	Odds ratio (95% Confidence Interval)	P value
Body mass index (kg/m^2^), <25/≥25	19.0 (2.8 – 127.0)	0.002
CA depth ratio, <0.370/≥0.370	13.7 (1.2 – 161.7)	0.038

## Discussion

Our study showed that a high BMI and larger upper abdomen independently influenced the risk of POPF in patients undergoing total gastrectomy with splenectomy for advanced gastric cancer. Previously in Japan, pancreaticosplenectomy had been routinely performed to dissect the lymph nodes along the splenic artery and around the splenic hilum in patients with gastric cancer in the upper third of the stomach [16]. However, many centers have recently reported the benefits of pancreas-preserving splenectomy [17-20]. Pancreas-preserving total gastrectomy with splenectomy was reported to be superior to total gastrectomy with pancreaticosplenectomy with respect to mortality, morbidity, and 5-year survival rate [4,8,21]. Although POPF developed in 49.7% of the patients who underwent total gastrectomy with pancreaticosplenectomy at our hospital, the present study showed that the incidence of POPF has decreased to 14.0% since the introduction of total gastrectomy with pancreas-preserving splenectomy in 2003 [22]. Although modifications of the surgical procedure and improved perioperative management have contributed to decreased morbidity and mortality, POPF remains a severe complication after total gastrectomy [5,23].

Obesity is a growing problem in developed countries and substantially increases the risks of morbidity and mortality associated with abdominal surgery [24-27]. BMI is considered a predictor of surgical outcomes in patients with different types of cancer, including colonic, breast, and endometrial malignancies [28-31]. Kodera et al reported that obesity increase the risk of surgical complications in patients who undergo distal gastrectomy with D2 lymphadenectomy [32]. Our study showed that a high BMI influences the risk of postoperative pancreas-related complications. This finding is consistent with the results of a previous study showing that being overweight increases the risk of surgical complications, including pancreatic fistula, in patients who undergo D2 dissection for gastric cancer [26].

Abdominal shape may also influence accessibility in patients with gastric cancer. Total gastrectomy with splenectomy is a more difficult procedure at deeper surgical sites because dissection is required around the esophageal hiatus or esophagojejunal anastomosis. Moreover, a large anterior-to-posterior abdominal wall diameter may make it difficult to dissect along the splenic artery or to mobilize the spleen in deep sites of the abdominal cavity. Lee et al. reported that obesity and abdominal shape at the umbilical level both influence the short-time outcomes of subtotal gastrectomy with D2 lymph node dissection in patients with gastric cancer [33]. In our study, we measured CAD and CATD to quantify upper abdominal shape, unlike previous studies [33]. We believe that a higher CA depth ratio requires a deeper surgical site. We found that upper abdominal shape as represented by CAD or CA depth ratio was related to the incidence of POPF, whereas body shape at the umbilicus was not. Tsukada et al. reported that accumulation of body fat is significantly associated with postoperative complications after elective gastric or colorectal surgery [27]. Seki et al. measured the visceral fat mass by using software to estimate fat volume, and examined the relation to operative time in patients with rectosigmoid cancer. They concluded that the amount of visceral fat was a more useful predictor of operative difficulty than was BMI [34]. Because we did not measure the amount of body fat in our study, the relations among upper abdominal shape, body fat amount, and POPF remain unclear.

Although, age, BMI, serum zinc level, hyperlipidemia, and comorbidity were significantly related to the incidence of POPF after pancreaticosplenectomy for advanced gastric cancer in our previous study [22], none of these factors, except for BMI, was positively associated with the incidence of POPF in this study. In contrast, we found that the shape of the upper abdomen significantly correlated with POPF. One of the reasons for the inconsistent results might be the difference in the operative procedures (pancreaticosplenectomy vs. pancreas-preserving splenectomy).

It is well-known that abdominal adiposity is strongly associated with increased incidence of diabetes mellitus (DM) which may also contribute to post-operative complication [4,35,36]. Russo et al. demonstrating that obesity and DM were independent predictors of surgical complication [37]. In our study, there were only 8 of 50 patients with DM. Among these patients, there was no significant difference of the incidence of POPF between the patients with and without DM.

Mathur et al. reported that fatty pancreas is risk of postoperative pancreatic leakage after pancreatoduodenectomy [38]. Kovanlikaya et al reported that there are positively correlated BMI and pancreatic fat content by magnetic resonance imaging [39]. Much visceral fat around pancreas is thought to make it hard to identify a border between pancreatic parenchyma and surrounding tissue. Therefore, there might be the risk for damaging the pancreatic substance without noticing. In our present study, no prospective data are available to correlate the texture of the pancreas. Therefore, future studies should be designed to capture this information and investigate the risk of POPF by analyzing the correlation between body shape, visceral fat around pancreas, and the texture of the pancreas.

It is thought that surgical procedure in a deep abdominal cavity such as left subphrenic space, dissection and ligation need particular skill. We think that it is easy for an experienced surgeon to imagine that the bleeding due to injury of the spleen is caused by immature surgical maneuver. It has been reported about utility of Ligasure™ in an operation of gastric cancer by randomized study [40]. We think that we can manipulate effectively and safety by using new surgical instrument (Ligasure™) in such a deep operation field, and we can perform safe maneuver recently. Therefore, the incidence of POPF might be low.

There are several potential limitations of our study. First, a major limitation of our study was the low-power statistics because of the small number of patients enrolled to this study. At our institution, although most patients with advanced gastric cancer are treated by total gastrectomy with splenectomy, most patients with early gastric cancer in the upper third of the stomach are treated by proximal gastrectomy or total gastrectomy without splenectomy. Hence it is difficult to collect large numbers of patients who underwent total gastrectomy with splenectomy. Splenectomy has been advocated to facilitate dissection of lymph nodes at the splenic hilum and along the splenic artery [4,41,42], and total gastrectomy without splenectomy has been performed for early gastric cancer which maneuver around pancreas is omitted by reduced range of lymphadenectomy. Therefore, we reviewed about incidence of a pancreas-related complication after splenectomy. Second, the proportion of patients with high BMI (BMI ≥ 25) in this study was low (16.0%). Therefore, the obtained results are not definitely conclusive, but our results suggest that caution is needed when performing total gastrectomy with splenectomy for gastric cancer in overweight patients. Third, a major limitation of our study was the low-power statistics because of the low incidence of POPF. Although mortality rates from gastrectomy complicated by pancreas-related abscess are lower in Japan than those reported in Western series [8,9], pancreas related abscess formation remains a strong factor in the mortality and morbidity rates in both Japanese and Western centers. Thus we think a large study will be necessary to obtain a definitive conclusion.

It does not have doubt that BMI is useful when we evaluate difficulty of operation for obese patient. However, in our result, there were not many cases with BMI high level. By contrast, there were some cases having high level of CA depth ratio in spite of low BMI. Therefore, we think it is important to measure upper abdominal body shape.

## Conclusion

In conclusion, our results indicate that surgical procedures such as total gastrectomy with splenectomy should be very carefully executed in obese patients or patients with a deep abdominal cavity to decrease the risk of postoperative pancreatic fistula. It is easy to measure the CAD and CATD at the level of the root of celiac artery by preoperative CT, and we can also do it retrospectively. Thus, CAD and CATD should be routinely evaluated in patients who undergo upper abdominal surgery particularly total gastrectomy with splenectomy. A worldwide study will be necessary to obtain a definitive conclusion.

## Competing interests

The authors declare that they have no competing interests.

## Authors' contributions

TS, HM, YN and SF carried out collection of data, and NY drafted the manuscript. TO and YR participated in the design of the study and performed the statistical analysis. CK and TI conceived of the study, and participated in its design and coordination and helped to draft the manuscript. All authors read and approved the final manuscript.
